# Gas–Liquid Two-Phase Flow Pattern Identification of a Centrifugal Pump Based on SMOTE and Artificial Neural Network

**DOI:** 10.3390/mi13010002

**Published:** 2021-12-21

**Authors:** Denghui He, Ruilin Li, Zhenduo Zhang, Shuaihui Sun, Pengcheng Guo

**Affiliations:** 1 Institute of Water Resources and Electric Power, Xi’an University of Technology, Xi’an 710048, China; lrl541@163.com (R.L.); 18234085789@163.com (Z.Z.); shs@xaut.edu.cn (S.S.); guoyicheng@xaut.edu.cn (P.G.); 2 State Key Laboratory of Eco-hydraulic in Northwest Arid Region of China, Xi’an University of Technology, Xi’an 710048, China

**Keywords:** gas–liquid flow, centrifugal pump, flow pattern identification, SMOTE algorithm, neural network

## Abstract

The accurate identification of the gas–liquid two-phase flow pattern within the impeller of a centrifugal pump is critical to develop a reliable model for predicting the gas–liquid two-phase performance of the centrifugal pump. The influences of the inlet gas volume fraction, the liquid phase flow rate and the pump rotational speed on the flow characteristics of the centrifugal pump were investigated experimentally. Four typical flow patterns in the impeller of the centrifugal pump, i.e., the bubble flow, the agglomerated bubble flow, the gas pocket flow and the segregated flow, were obtained, and the corresponding flow pattern maps were drawn. After oversampling based on the SMOTE algorithm, a four-layer artificial neural network model with two hidden layers was constructed. By selecting the appropriate network super parameters, including the neuron numbers in the hidden layer, the learning rate and the activation function, the different flow patterns in the centrifugal pump impeller were identified. The identification rate of the model increased from 89.91% to 94.88% when the original data was oversampled by the SMOTE algorithm. It is demonstrated that the SMOTE algorithm is an effective method to improve the accuracy of the artificial neural network model. In addition, the Kappa coefficient, the Macro-F1 and the Micro-F1 were 0.93, 0.95 and 0.95, respectively, indicating that the model established in this paper can well identify the flow pattern in the impeller of a centrifugal pump.

## 1. Introduction

As important energy conversion and fluid transportation equipment, centrifugal pumps are widely used in the petrochemical, coal chemical, and oil and gas extraction fields [1]. In practical engineering applications, gas–liquid two-phase flow is frequently encountered, reducing the performance of the pump [2,3]. The increase of inlet gas volume not only affects the pressure increment and efficiency of a centrifugal pump, but also produces surging and even gas locking of the pump, which will endanger the stability of flow system and reduce the service life of the pump [4,5]. Therefore, the prediction of pump performance under the gas–liquid two-phase flow condition is of great significance. Studies show that pump performance is closely related to the flow pattern in the pump [6]. Thus, the correct identification of the flow pattern in the pump is essential for developing prediction models to analyze the flow characteristics and performance of the pump.

Currently, two methods are available for the identification of two-phase flow patterns. The first one is the direct measurement method, which determines the flow pattern from the flow image. Visual inspection, high speed photography [6,7,8,9,10] and tomographic imaging are several typical methods used [11,12]. He et al. [6,10] investigated the flow pattern in the impeller of a centrifugal pump using high-speed camera technology and obtained four different flow patterns. The effects of the inlet gas volume fraction (IGVF), liquid flow rate and rotational speed on the distribution of the gas–liquid phase in the impeller was analyzed, as were the pump pressure increment and efficiency. Verde et al. [8] observed four typical flow patterns using the high-speed camera technique and determined that the centrifugal pump performance variation is related to the gas–liquid two-phase flow characteristics in the pump. In addition, they obtained a flow pattern versus pump performance graph under different operating conditions. Schäfer et al. [11] obtained distribution images of high gas content in the impeller by the gamma-ray scanning technique, and determined the effect of inlet flow conditions on the performance of centrifugal pumps.

The second type is the indirect measurement method, which works by measuring the fluctuating signals reflecting the flow characteristics and then processing them for analysis. Pressure, differential pressure, gas volume fraction and void fraction are several parameters that are frequently employed. Unfortunately, the indirect method is mainly applied for identification of the flow patterns in pipes and channels [13,14,15,16,17,18], while few studies have been reported concerning centrifugal pumps. Li et al. [14] identified flow patterns through the pressure characteristics of each branch of the riser and the probability density function (PDF) of the differential pressure. Yin et al. [16] carried out a multi-scale marginal spectral entropy analysis on the differential pressure signal, which can distinguish the four flow regimes in the beam channel, macroscopically. Du et al. [18] analyzed the wave signals measured in the vertical pipeline by using the adaptive optimal kernel time-frequency algorithm (AOK TFR), and distinguished different flow patterns and their complex dynamic behaviors. Sun et al. [19] also identified gas–liquid two-phase flow patterns after analyzing the differential pressure signal in the horizontal Venturi based on the time-frequency signal processing method of the adaptive optimal kernel (AOK). Euh et al. [20] carried out a wavelet analysis on the void fraction signal measured in the vertical channel and obtained the identification criteria of different flow patterns by calculating the effective local wavelet energy and scale in the time-frequency diagram.

In recent years, machine learning has shown excellent characteristics in solving complex problems in engineering applications and scientific fields, which provides a new idea for flow pattern recognition of centrifugal pump under gas–liquid two-phase conditions [21,22]. As an important method to realize machine learning tasks, artificial neural networks mainly include algorithms such as Back Propagation (BP), Multilayer Perceptron (MLP), Radial Basis Function (RBF), Probabilistic Neural Network (PNN) and Convolutional Neural Network (CNN). Many studies have been published on two-phase flow pattern identification based on artificial neural networks for their good classification ability [23,24,25,26]. Lin et al. [23] constructed a 5-layer BP neural network using the superficial velocities of water and air and the channel inclination angle as inputs. They identified six flow patterns in channels with different inclination angles and found that the results matched the Barnea unified model well. Xu et al. [24] realized the flow pattern recognition of long-distance riser system based on BP neural network by extracting the pressure signal feature and reducing the dimension of the principal component analysis (PCA). The influence of the pressure signal and signal length at different positions on the recognition rate were also analyzed. They found that a higher recognition rate can be obtained when the differential pressure is used as the input. Rosa et al. [26] investigated the flow pattern in a vertical channel based on the void fraction and the relevant probability density function. They also compared the identification results of flow patterns using the MLP, RBF and PNN algorithm models. Abbagoni et al. [27] classified different flow patterns of gas–liquid two-phase flow by ultrasonic signal and artificial neural network, and compared the recognition rate of models under different feature extraction methods. Ghosh et al. [28] used the statistical parameter training model of a probe signal and three different artificial neural network models to identify counter-current gas–liquid two-phase flow.

The present work aims to develop a prediction model based on an artificial neural network to identify the flow pattern in the impeller of the centrifugal pump. First, experiments were conducted on the gas–liquid two-phase flow pattern of the centrifugal pump. The influence of the inlet gas volume fraction, the rotational speed and the liquid flow rate on the flow pattern in the impeller were obtained, and thus the basic database was established. Then, the SMOTE algorithm was used to over sample a few samples and the suitable network hyperparameters were selected by keeping the number of sample classes balanced, after which an artificial neural network-based flow pattern identification model for centrifugal pumps was constructed. Finally, the identification results of four flow patterns were analyzed and evaluated.

## 2. Visualization Experiment on Gas–Liquid Two-Phase Flow

### 2.1. Experimental System

**Figure 1:**
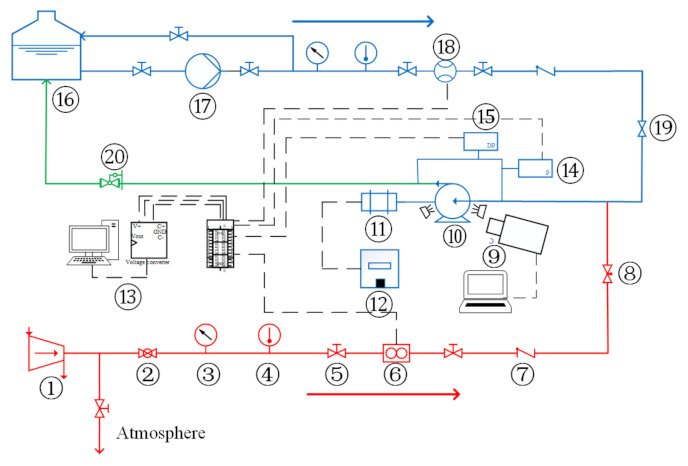
Schematic diagram of visual experimental system for gas–liquid two-phase flow of centrifugal pump: 1-air compressor; 2-ball valve; 3-pressure meter; 4-thermometer; 5-globe valve; 6-gas flowmeter; 7-check valve; 8-needle valve; 9-high speed camera system; 10-test pump; 11-electric motor; 12-frequency converter; 13-data acquisition system; 14-pressure transmitter; 15-differential pressure transmitter; 16-water storage tank; 17-multistage centrifugal pump; 18-water mass flowmeter; 19-regulating valve; 20-back pressure regulator (gate valve).

Figure 1 illustrates the gas and liquid two-phase flow experiment apparatus of the centrifugal pump used in this study. The working fluids were tap water and compressed air. After being compressed by the air compressor, the air was regulated by the regulating valve, dehumidified by the filter, and then measured by the gas laminar mass flowmeter (uncertainty ± 0.5%). Afterwards, it was injected into the liquid phase circuit through needle valves. The liquid phase was pumped to the liquid phase mass flowmeter (uncertainty ± 0.2%) by a multistage centrifugal pump. The gas and liquid mixed at 3 times the diameter of the impeller inlet upstream of the test pump inlet, and then entered the test pump. The gas–liquid mixture in the experimental pump outlet entered the gas–liquid separation device for gas and liquid separation, and then the air was discharged into the atmosphere and the water entered the water tank for recycling. The gas and liquid phase flow rates, the inlet pressure, the temperature, and the pressure increment between the pump inlet and outlet were measured. The acquisition program was developed based on Labview 2015 (National Instruments, Austin, TX, USA), and the data acquisition device was an NI USB-6229 high-speed data acquisition board. The parameters of the main measurement devices used in the experiments are shown in Table 1.

Table 2 shows the main parameters of the test centrifugal pump, which was manufactured using polymethyl methacrylate (PMMA) to observe the flow pattern. The flow pattern of gas–liquid two-phase flow in the impeller of the centrifugal pump was filmed with a Photron FASTCAM Mini AX 200 (Tokyo, Japan) high-speed camera.

### 2.2. Experimental Scheme

In this experiment, the pump inlet and outlet pressure increments (∆*P*) was measured while gradually increasing the pump inlet gas volume fraction (IGVF) at a certain pump rotational speed (*N*) and liquid phase flow rate (*Q*_L_). The parameters of the experimental conditions are shown in Table 3 (*Q*_BEP_ in the table is the liquid flow rate corresponding to the optimal efficiency point at the corresponding rotational speed). The pressure at the pump inlet and the pressure increment at the inlet and outlet were measured by pressure and differential pressure sensors, respectively (uncertainty ± 0.075%). For each test condition, when the pump flow was stable, the data acquisition with the acquisition frequency of 500 Hz and the time of 60 s began.

### 2.3. Gas Liquid Two-Phase Flow Pattern in Centrifugal Pump

**Figure 2:**
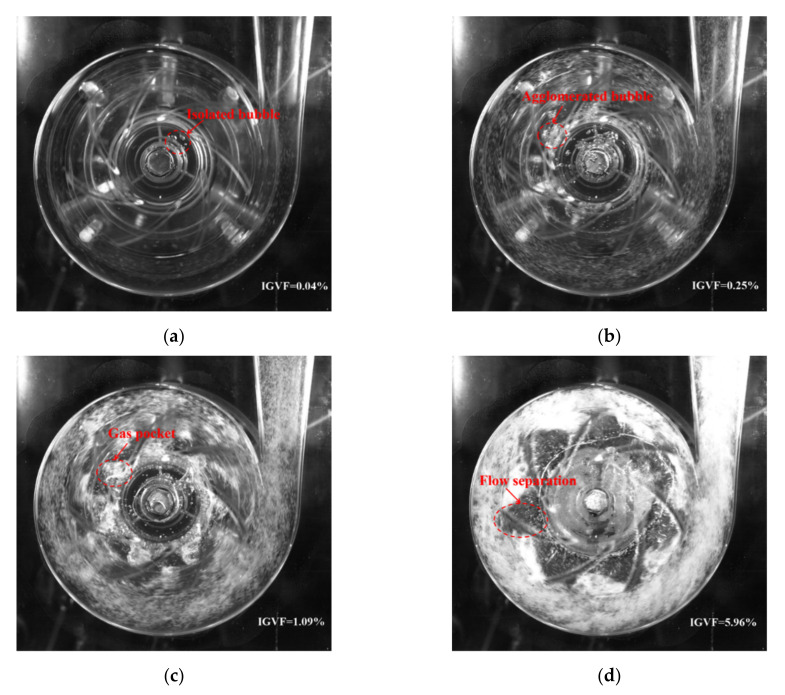
Flow images of four flow patterns in impeller (*N* = 1200 rpm, *Q_L_* = 5.7 m^3^/h). (**a**) Bubble flow (BF); (**b**) Agglomerated bubble flow (ABF); (**c**) Gas pocket flow (GPF); (**d**) Segregated flow (SF).

Figure 2 shows four typical flow patterns, i.e., bubble flow (BF), agglomerated bubble flow (ABF), gas pocket flow (GPF) and segregated flow (SF), in the impeller at different inlet gas volume fraction obtained by the high-speed camera. The rotational speed *N* = 1200 rpm and the liquid phase flow rate *Q*_L_ = 5.7 m^3^/h. The features of these flow patterns have been well described in references [3,6,7,8,9,10].

**Figure 3:**
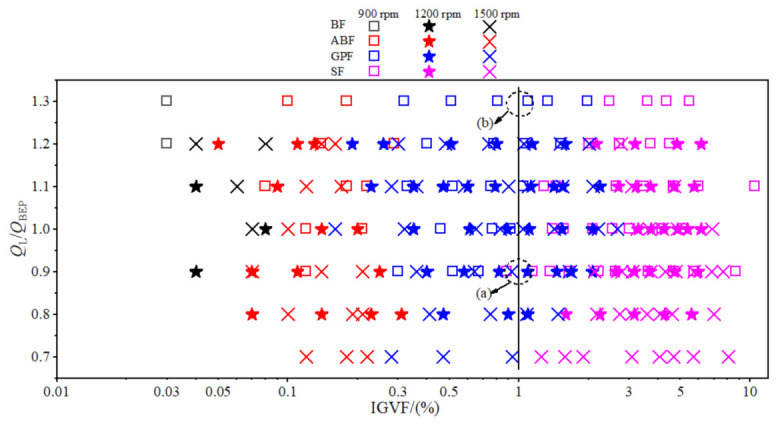
Flow pattern map of centrifugal pump.

Figure 3 shows the flow pattern under different liquid flow rate and inlet gas volume fraction with the rotational speed of 900 rpm, 1200 rpm and 1500 rpm. We found that the BF pattern only appeared under the condition of extremely low IGVF (the black data points in Figure 3) and no obvious bubble accumulated in the impeller (Figure 2a). With the increase of IGVF, the BF pattern converted to the ABF pattern (the red data points in Figure 3); the bubbles at the inlet of impeller channel merged and agglomerated, resulting in the generation of large bubbles (Figure 2b). The flow pattern changed from ABF to GPF (the blue data points in Figure 3) when the IGVF continued increasing; the gas and liquid mixed violently at the inlet of the impeller (Figure 2c), resulting in the increase of flow loss. As the IGVF further increased, the flow pattern in the impeller changed to SF (the purple data points in Figure 3). At this time, the gas phase and the liquid phase in the impeller were separated, a stable and slender large bubble stagnated in the middle of the impeller channel, and the front and rear cover plates of the impeller were covered with a liquid film layer, forming a flow form similar to the annular flow in the pipeline (Figure 2d) [6,7].

In addition, it can be seen from Figure 3 that the flow pattern was affected by the liquid phase flow rate. The flow pattern in the impeller was an SF pattern when the liquid phase flow *Q*_L_/*Q*_BEP_ was 0.9 under *N*= 900 rpm and IGVF = 1% (see (a) in Figure 3). When the *Q*_L_/*Q*_BEP_ increased to 1.3, the flow pattern in the impeller changed to GPF (see (b) in Figure 3). At the same time, rotational speed also affected the flow pattern in the impeller. For instance, as the rotational speed increased from 900 rpm to 1200 rpm or 1500 rpm, the flow pattern changed from the SF to GPF under *Q*_L_/*Q*_BEP_ = 0.9 and IGVF = 1% (shown in (a) of Figure 3). It is suggested that the accumulation and retention of gas in the impeller can be reduced by increasing the liquid flow and rotational speed, thus improving the gas carrying capability of the pump. Therefore, we concluded that the flow pattern in the centrifugal pump was related to the inlet gas volume fraction, the liquid phase flow rate, the pump rotational speed and the pressure increment. Because of the complexity of the pump flow pattern, the transition boundary between the regimes is still difficult to quantitatively determine [2]. The data of the present experiment are available in the Supplementary Materials as Table S1: Experiment data of the flow pattern in the impeller.

## 3. Model Building and Training

**Figure 4:**
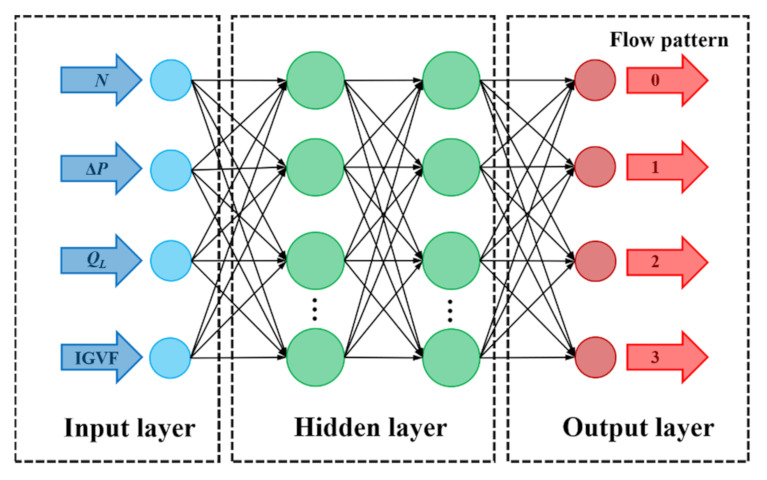
Neural network structure.

A multi-layer BP artificial network model was used to classify and predict the gas–liquid two-phase flow pattern of the centrifugal pump. As a multilayer feedforward network with error back propagation, a BP neural network possesses strong nonlinear mapping approximation ability. It is one of the most widely used neural networks at present. It consists of the input layer, the hidden layer and the output layer, whose output results are propagated forward and whose errors are back-propagated. Figure 4 shows the network structure employed in this study. The inputs are the inlet gas volume fraction (IGVF), the liquid phase volume flow rate (*Q*_L_), the pump rotational speed (*N*) and the pressure increment (Δ*P*). The output is the probability value corresponding to four flow patterns, which are denoted as 0, 1, 2 and 3, respectively.

### 3.1. SMOTE Oversampling

The sample numbers of the four flow patterns are shown in Table 4. The BF only appeared when the IGVF was extremely low. Because of the limitation of the experimental system, only 9 samples of BF patterns and 37 samples of ABF patterns were available in this study. The imbalance rate of the BF pattern and the ABF pattern to the SF pattern was 10 and 2.4, respectively. Under this situation, the model will pay more attention to the SF pattern, which will degrade into the classification performance of BF pattern and ABF pattern. Thus, the imbalance rate problem should be resolved to achieve good recognition accuracy.

The SMOTE algorithm can be employed to resolve the imbalance rate problem of the experimental data and enhance the data. The algorithm is an oversampling technique to synthesize new samples after analyzing the samples with a small number. Compared with the random oversampling technique which is easy to make the model be over fit, the SMOTE algorithm samples the feature space, so its accuracy is higher than that of the traditional sampling method [29,30]. The algorithm is shown as follows:

(1) For sample *a* with low proportion in the category, the Euclidean distance *s* to sample *b* in the category is calculated by Equation (1):

(1) $$s=\sqrt{\sum_{m=1}^{4}{\left(a_{m}-b_{m}\right)}^{2}}$$

(2) Taking point *a* as the center, *k* adjacent samples are selected according to Euclidean distance *s* to obtain their *k* proximity.

(3) A sample point *c* is randomly selected from the nearest neighbors of *k*, and its linear interpolation *d* to the original sample point *a* are calculated according to Equation (2). *d* is a minority sample point similar to *a*;

(2) $$d=a+\delta \left(c-a\right)$$

where *δ* is a random number between 0 and 1.

(4) Set the sampling scale and repeat the above interpolation process. Then the synthesized samples are added to the original data set.

### 3.2. Selection of Network Super Parameters

The structure of a neural network has an important influence on the prediction results. A series of hyperparameters, such as the number of neurons in the hidden layer, the activation function, etc., need to be determined to build the network model.

#### 3.2.1. Selection of Number of Hidden Layers

When the number of hidden layers is one, various functions containing continuous mappings from one finite space to another finite space can be fitted; when a number of hidden layers are double-layer, various precision decision boundaries can be represented with appropriate activation functions, and smooth mappings of various precision can be fitted. In order to make the network model effectively learn the flow characteristics of gas–liquid two-phase flow and accurately predict various flow patterns under different working conditions, a double-layer hidden layer was selected for flow pattern identification in this paper.

#### 3.2.2. Number Selection of Hidden Layer Neurons

Generally, there is no general solution to determine the number of nodes. If the number of hidden layer nodes is too small, the network may not be trained at all or the network performance will be very poor; if the number of hidden layer nodes is too large, the system error of the network can be reduced. However, the network training time will be much longer on the one hand, and on the other hand, the training may fall into local minima and cannot achieve the optimal point, which is also the essential reason for over fitting during training. If a small network with few nodes is selected, the loss value is extremely high, even though the network is easy to converge to local minima. While a large, multi-node network is selected, the loss value is small and more local minima can be found. In the present study, 512 neurons were ultimately selected for network training.

#### 3.2.3. Selection of Activation Function

For the hidden layer, the Relu activation function was selected, as it can overcome the gradient disappearance problem and greatly reduce the training time of the network [23]. For the output layer, the softmax activation function [31,32] is chosen to output the probability values predicted by the network for the four flow patterns. The sum of the four probability values equals one, and the flow pattern corresponding to the maximum probability value is the flow pattern predicted by the network.

#### 3.2.4. Learning Rate

When the gradient decreases, it is necessary to specify a learning rate as a control factor for the weight update step, for which learning rates of 0.01, 0.001 and 0.0001 are often used. In this study, the exponential attenuation of learning rate is adopted. A large learning rate was used in the early training stage to make the network converge quickly. The learning rate was gradually reduced with the increase of the number of iterations, so as to better make the network converge to the optimal solution.

#### 3.2.5. Iteration Number

Too few iterations will make the network under fit, and too many iterations will make the network over fit. The number of iterations suitable for the network model should be reasonably selected. The Early Stopping method was used here to calculate the correct rate on the training set after each iteration. When the number of iterations exceeds a certain range, the accuracy of the model remains unchanged or its improvement becomes too small. We worked to avoid over fitting and to improve the generalization ability of the network.

#### 3.2.6. Regularization Parameter

In the training process of the network, L2 regularization was added to the Early Stopping method to avoid the over-fitting of the network. L2 regularization controls the complexity of the model by adding a penalty term to the original loss function to penalize a model with high complexity [33], as shown in Equation (3):

(3) $$\tilde{L}=L+\lambda \sum_{i}\omega_{i}{}^{2}$$

where *L* is the training error, i.e., the loss function, $\tilde{L}$ is the training error after regularization, *λ* is an adjustable regularization parameter used to control the strength of regularization and $\sum_{i}\omega_{i}{}^{2}$ is the sum of the squares of weights, which needs to meet Equation (4).

(4) $$\sum_{i}\omega_{i}{}^{2}\leq C$$

where *C* is the upper limit of the sum of the squares of the weights, that is, the sum of the squares of weights of the network cannot exceed parameter *C*. Thus, after adding L2 regularization, the goal of the network is to minimize the training error $\tilde{L}$ within this constraint.

### 3.3. Model Building Process and Steps

**Figure 5:**
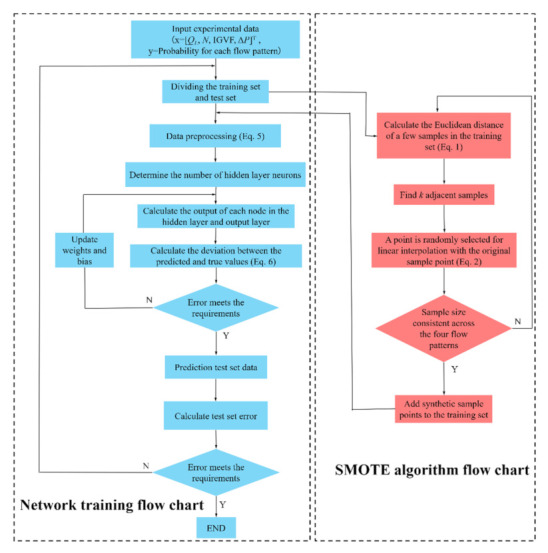
Model training flow chart.

The model training flow chart with SMOTE oversampling is shown in Figure 5. The specific construction and training process of the model is as follows:

Step 1: determination of input and output parameters. In order to make the network model better classify and predict the four flow patterns of gas–liquid two-phase flow, the inlet gas volume fraction (IGVF), the pressure increment (Δ*P*), the rotational speed (*N*) and the liquid phase flow (*Q*_L_) were selected as inputs. The flow patterns divided according to observations by the high-speed camera are used as the outputs, which was taken as the original data set.

Step 2: dividing the training set and test set. A total of 218 sample points were obtained from the experiment. In order to obtain a reasonable network model, the data set needs to be divided into training and test sets. In the present work, 80% of the experimental data (174 samples) were are selected as the training set, and the remaining 20% (44 samples) constituted the test set.

Step 3: SMOTE oversampling. Since imbalance in the distribution of the four flow patterns can lead to a significant reduction in the classification performance of the model, the oversampling of the samples with a small number in the training set is required after dividing the training and test sets. Table 5 shows the number of samples in the training set of each flow pattern before and after the data were enhanced by SMOTE.

Step 4: data preprocessing. Before the data is input to the network for training, the input and output parameters need to be feature extracted and normalized. For the output parameters, the labels 0, 1, 2 and 3 are used to represent the BF, ABF, GPF and SF patterns, respectively. For the input parameters, the Zero-mean normalization (Z-score standardization) is required. Four input quantities, including inlet gas volume fraction (IGVF), liquid phase flow *Q*_L_, pump rotational speed *N* and pressure increment Δ*P* were standardized, respectively. The mean value and the standard deviation were 0 and 1, respectively, after processing. The standardized equation is shown in Equation (5):

(5) $$\hat{x_{ij}}=\left(x_{ij}-\bar{x_{i}}\right)/\sigma_{i}$$

where *x_ij_* is the *j*th original value of input feature *i*, $\bar{x_{i}}$ is the mean value of characteristic *i*, $\sigma_{i}$ is the standard deviation of characteristic *i*. After standardization, if the original value of the data is greater than its mean value, positive standardized data will be obtained. Otherwise, negative standardized data will be obtained.

Some sample points after data preprocessing are shown in Table 6.

Step 5: neural network training. The number of network layers adopts a four-layer network structure, that is, the neural network includes an input layer, an output layer and two hidden layers. The number of nodes in each layer was 4, 512, 512 and 4, respectively.

Step 6: deviation calculation. Cross-entropy loss was used to test the deviation between the predicted value of the model and the real value. It is defined by Equation (6):

(6) $$L=\frac{1}{M}\sum_{i}^{M}L_{i}=-\frac{1}{M}\sum_{i}^{M}\sum_{t=1}^{m}y_{it}\log\left(p_{it}\right)$$

where *M* is the number of samples in the training set, *L_i_* is the error of the *i*th training sample, *m* is the number of categories, *m* = 4; *p_it_* represents the probability that the *i*th sample is predicted as category *t*, *y_it_* is a symbolic function, if the true category of sample *i* is *t*, it equals 1, otherwise it is 0

Step 7: model verification. The untrained test data sets were are used to evaluate the performance of the network model.

### 3.4. Network Iteration Curve

**Figure 6:**
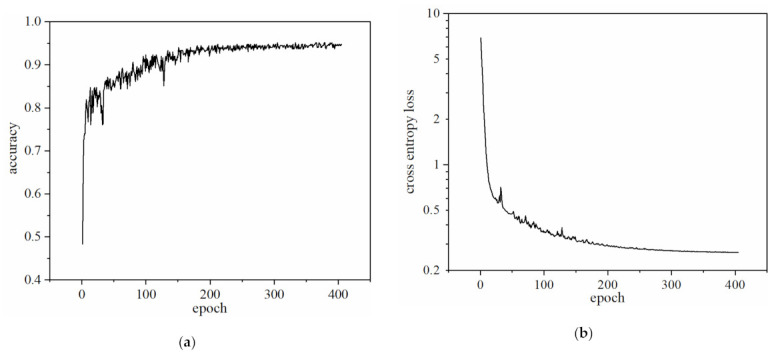
Iterative curve of network on training set. (**a**) Model accuracy curve; (**b**) Model cross entropy loss function curve.

Accuracy was selected as the evaluation function of the network, as shown in Figure 6a. With the increase of the iteration numbers (epoch), the accuracy shows an upward trend and finally tends to stabilize. When the accuracy barely increases with iterations, the network training comes to an end. The accuracy of the network model on the present training set was 0.95. Figure 6b shows the iterative curve of the cross-entropy loss function. With the increase of the iteration numbers, the loss value of the network continues to decline and finally tends to stabilize.

## 4. Discussion

### 4.1. Comparison of Recognition Rate

In order to evaluate the effects of the SMOTE oversampling on the network performance, the classification performance before and after data enhancement was compared on each sample set, and in particular on the performance on the test set.

Table 7 shows comparisons of the identification results of four flow patterns between the original data set and the enhanced data set. It can be seen that the training data of the BF that the ABF and the GPF increased from 6, 30 and 66 to 72 after data enhancement by SMOTE, which was equal to the increase to the SF pattern. The model failed to recognize the BF pattern in the original data set, including the training set and the test set. When the model was trained by the enhanced data, we found that no misjudged data in the training set for the BF pattern and only one misjudged datum in the test set. The identification rate of the BF pattern in the test set was 66.67%. For the ABF pattern, the identification rate in the test data also increased from 71.43% to 100%. In addition, the identification rate in the test data of the GPF pattern was also slightly raised from 82.36% to 88.24%. The model performed best for the SF pattern among the four flow patterns. It is concluded that the SMOTE oversampling technique is beneficial to predict datasets with large imbalance rates. The generalization and robustness of the model based on the artificial neural network can be improved significantly when combined with the SMOTE technique.

**Figure 7:**
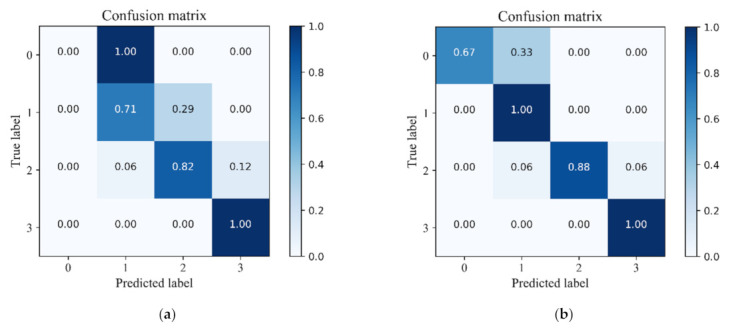
Confusion matrix of model of the test set. (**a**) Before data enhancement; (**b**) After data enhancement.

As shown in Figure 7, the confusion matrix plot based on the classification results of the model on the test set for the four flow patterns also displayed the identification rate. The horizontal and vertical coordinates in the figure are the predicted and actual flow patterns, respectively. The color in the legend denotes the identification rate. The results demonstrated that the prediction performance of the model was improved when it was trained by the enhanced data.

Note that part of the GPF samples were still misjudged as the ABF and the SF, no matter whether or not the training data was enhanced by the SMOTE oversampling technique. This was mainly due to the GPF pattern being the transition flow pattern between the ABF pattern and the SF pattern. GPF shows strong unstable characteristics, at which the surging phenomenon of the pump tends to occur [34]. It is suggested that more characteristic signals are required to further improve the prediction performance of the model on the GPF pattern.

### 4.2. Receiver Operating Characteristic (ROC) Analysis

In order to further evaluate the classification performance of the model for each flow pattern, a Receiver Operating Characteristic curve (ROC curve) is introduced for analysis. The ROC curve is drawn based on the results of different judgment criteria, such as normal, roughly normal, suspicious, roughly abnormal and abnormal. The horizontal and vertical coordinate are the true positive rate (sensitivity) and the false positive rate (specificity), respectively. In the ROC curve, the closer the curve is to the upper left corner and the larger the Area Under the Curve (AUC, the minimum value is 0.5), the better the classification performance of the model.

**Figure 8:**
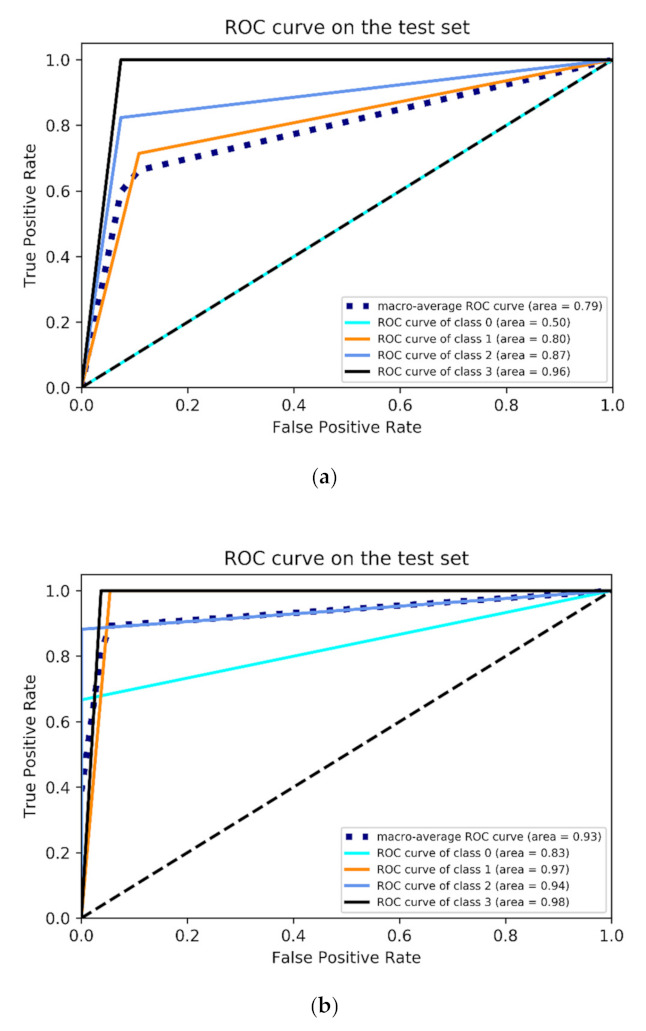
ROC curve of model on the test set. (**a**) Before data enhancement; (**b**) After data enhancement.

Figure 8 shows the ROC curve of the model on the test set before and after data enhancement. For the BF pattern (class 0), the AUC was 0.5 before the data were enhanced using the SMOTE (Figure 8a), which means that the authenticity of BF classification by the model was 0; while the AUC increased to 0.83 after data enhancement (Figure 8b), which shows a significant improvement in the classification performance of the model. Meanwhile, the ROC curves of the other three flow patterns also shifted to the upper left corner after data enhancement and the AUC was much higher than before data enhancement.

In addition, the macro-average ROC curve was also employed to evaluate the overall classification performance of the model. The AUC of the macro-average ROC curve before and after data enhancement was 0.79 and 0.93, respectively. The ROC curve after SMOTE algorithm processing was much closer to the upper left corner, which indicates that the overall classification performance of the model improved significantly.

### 4.3. Model Evaluation

In addition to the identification rate *P*_0_, another three idices, i.e., Kappa coefficient, Macro-F1 and Micro-F1, were introduced to further evaluate the model performance.

The Kappa coefficient is used in statistics to assess consistency, which is defined by Equation (7):

(7) $$\mathrm{Kappa}=\frac{P_{0}-P_{e}}{1-P_{e}}$$

where *P*_0_ and *P_e_* are calculated by Equations (8) and (9)

(8) $$P_{0}=\frac{\sum_{i=1}^{m}TP_{i}}{Z}$$

(9) $$P_{e}=\frac{\sum_{i=1}^{m}\left[\left(TP_{i}+FN_{i}\right)\times \left(TP_{i}+FP_{i}\right)\right]}{Z^{2}}$$

where *TP_i_*, *FP_i_* and *FN_i_* are the number of True Positives, False Positives, and False Negatives in category *i*, respectively, *m* is the number of categories, *m* = 4, *Z* is the total number of samples.

The F1-score is a measure of accuracy in a dichotomous classification problem, and is used to measure the accuracy of uneven distribution data. It takes into account both the precision (*p*) and recall (*r*) of the classification model. In multi-classification problems, there are two ways to calculate the F1-score of a model, which are Macro-F1 and Micro-F1. In the uneven distribution sample set, for an F1 value, Macro-F1 focuses on the category with the fewest samples, while Micro-F1 focuses more on the category with the most samples. They are calculated by Equations (10)–(15):

(10) $$\mathrm{Macro}-F1=\frac{1}{m}\sum_{i=1}^{m}\frac{2p_{i}r_{i}}{p_{i}+r_{i}}$$

(11) $$p_{i}=\frac{TP_{i}}{TP_{i}+FP_{i}}$$

(12) $$r_{i}=\frac{TP_{i}}{TP_{i}+{FN}_{i}}$$

(13) $$\mathrm{Micro}-F1=\frac{2p_{\mathrm{Micro}}r_{\mathrm{Micro}}}{p_{\mathrm{Micro}}+r_{\mathrm{Micro}}}$$

(14) $$p_{\mathrm{Micro}}=\sum_{i=1}^{m}\frac{TP_{i}}{TP_{i}+FP_{i}}$$

(15) $$r_{\mathrm{Micro}}=\sum_{i=1}^{m}\frac{TP_{i}}{TP_{i}+{FN}_{i}}$$

Table 8 shows the evaluation index of the model on the training set, test set and total data set before and after data enhancement. It is can be seen that, before data enhancement, the identification rate *P*_0_ of the model on the training set was 91.95%, but it was only 81.82% on the test set, which means that the generalization of the model is poor. After data enhancement, the identification rate of the model was higher than 93% for all three sample sets. The generalization of the model was significantly improved. In addition, the Kappa coefficient, Macro-F1 and the Micro-F1 were higher than 0.9 after data enhancement, which also shows the good classification performance of the model.

For the model performance on the test set before data enhancement, the Macro-F1 and the Micro-F1 were 0.6 and 0.82 respectively, indicating that the model performed poorly in the category with few samples (BF pattern) for classification. After data enhancement, the Macro-F1 and the Micro-F1 increased to 0.9 and 0.93, respectively, which demonstrates that the model could accurately identify all types of flow pattern samples. The classification performance of the model was significantly improved.

## 5. Conclusions

In this paper, the flow characteristics of centrifugal pump under gas–liquid two-phase conditions were experimentally investigated and the corresponding flow pattern map was obtained. The two-phase flow pattern identification model of a centrifugal pump based on the SMOTE algorithm and a multilayer BP artificial neural network was constructed by determining the network hyperparameters, such as the number of layers and neurons, the iteration number and the learning rate. The classification performance of the model was also analyzed and evaluated.

Four typical flow patterns, i.e., Bubble Flow, Agglomerated Bubble Flow, Gas Pocket Flow and Segregated Flow, in the impeller of centrifugal pump were observed with the increase of the inlet gas volume fraction. The transformation of the flow pattern in the impeller was delayed and the gas carrying capability of the pump was improved by increasing the liquid phase flow and the pump rotational speed. The uneven distribution problem of samples on the training set was solved by data enhancement using the SMOTE algorithm. The overfitting of the model to the samples with a small number was avoided, which reduced data misclassification in the test set and significantly improved the identification rate of the four flow patterns, especially the Bubble Flow pattern.

A discrete flow pattern such as the Segregated Flow can be well identified because it has obvious, characteristic features. However, for the flow data in the transition region of two flow patterns, the input parameters used in the present study were inadequate to characterize their features. Therefore, more information (e.g., the probability density function (PDF) and power spectral density (PSD) of the differential pressure signals) to reflect the characteristics of the flow patterns in the transition region are required for further investigation.

## Figures and Tables

**Table 1:** Measurement devices employed in the experiments.

Device	Measurement Range	Uncertainty	Manufacturer
Gas laminar flowmeter	0–1 L/min	± 0.5 % (0.25% R.^(1)^+ 0.25% F.S.^(2)^)	Yidu Intelligence^®^, Xi’an, China
	0–20 L/min	± 0.5 % (0.25% R.+ 0.25% F.S.)	
Water Coriolis mass flowmeter	0–5500 kg/h	± 0.2 % R. (10:1)	Sincerity^®^, Beijing, China
	0–30,000 kg/h	± 0.2 % R. (10:1)	
Temperature sensor	0–60 °C	± 0.15 °C (R.)	Xi’an Instruments Factory^®^, Xi’an, China
Pressure sensor	0–0.5 MPa	± 0.5 % F.S.	KELLER^®^, Switzerland
Differential pressure sensor	0–6.22 kPa 0–16.25 kPa 0–62.5 kPa 0–150 kPa	± 0.075 % F.S.	Emerson Process Management^®^, St. Louis, MO, USA
Data acquisition board	48 input channels 80 kS/s	16 bits	National Instrumentation^®^, Austin, TX, USA

Note: (1) R. is Reading error; (2) F.S. is Full Scale error.

**Table 2:** Parameters of the centrifugal pump.

Flow Rate *Q_d_* (m^3^/h^)^	Total Head *H_d_* (m)	Rotational Speed *n* (rpm)	Diameter of impeller Inlet *D*_in_ (mm)	Diameter of Impeller *D*_I_ (mm)	Width of Blade Outlet *b* (mm)	Diameter of Pump Outlet *D*_out_ (mm)	Average Diameter of Volute Cutwater *D_cw_* (mm)	Number of Blades *Z*
17.0	17.0	3000	50	115	9.2	40	131.5	7

**Table 3:** Parameters of experimental conditions.

Rotational Speed, *N*/(rpm)	*Q*_L_ */Q*_BEP_	Inlet Gas Volume Fraction, IGVF/(%)
900	0.9–1.3	0–10.55
1200	0.8–1.2	0–6.12
1500	0.7–1.2	0–8.06

**Table 4:** Description of experimental data set.

Flow Pattern	Number of Samples
BF	9
ABF	37
GPF	83
SF	89

**Table 5:** Description of training data set before and after data enhancement.

Flow Pattern	Number of Samples	
	Original Data	After SMOTE Algorithm Processing
BF	6	72
ABF	30	72
GPF	66	72
SF	72	72

**Table 6:** Some sample points after data preprocessing.

$\hat{x_{IGVF}}$	$\hat{x_{∆P}}\;$	$\hat{x_{N}}\;$	$\hat{x_{Q_{L}}}\;$	Label
−0.701353	−0.688075	−1.312674	−0.265161	0
−0.604578	1.703384	1.206349	−0.027790	1
0.045971	−0.109064	−0.053163	0.446954	2
2.223426	−1.660258	−1.312674	−0.265161	3

**Table 7:** Comparison of identification results.

Sample Set	Flow Pattern	Number of Samples		Correct Number of Samples		Recognition Rate *P*_0_	
		Original Data Set	Enhanced Data Set	Original Data Set	Enhanced Data Set	Original Data Set	Enhanced Data Set
Training set	BF	6	72	0	72	0	100%
	ABF	30	72	28	65	93.33%	90.28%
	GPF	66	72	62	66	93.94%	91.67%
	SF	72	72	70	71	97.22%	98.61%
Test set	BF	3	3	0	2	0	66.67%
	ABF	7	7	5	7	71.43%	100%
	GPF	17	17	14	15	82.36%	88.24%
	SF	17	17	17	17	100%	100%
Total data set	BF	9	75	0	74	0	98.67%
	ABF	37	79	33	72	89.19%	91.14%
	GPF	83	89	76	81	91.57%	91.01%
	SF	89	89	87	88	97.75%	98.88%

**Table 8:** Evaluation indexes of the model on different sample sets before and after data enhancement.

Index	Training Set		Test Set		Total Data Set	
	Before Enhancement	After Enhancement	Before Enhancement	After Enhancement	Before Enhancement	After Enhancement
Identification rate, *P*_0_	91.95%	95.14%	81.82%	93.18%	89.91%	94.88%
Kappa coefficient	0.88	0.94	0.72	0.90	0.84	0.93
Macro-F1	0.69	0.95	0.60	0.90	0.67	0.95
Micro-F1	0.92	0.95	0.82	0.93	0.90	0.95

## Data Availability

The data presented in this study are available in supplementary materials.

## Supplementary Materials

The following are available online at https://www.mdpi.com/article/10.3390/mi13010002/s1, Table S1: Experiment data of the flow pattern in the impeller.

## References

[1] Gülich J.F. (2020). Centrifugal Pumps.

[2] Zhu J., Zhang H.Q. (2018). A review of experiments and modeling of gas-liquid flow in electrical submersible pumps. Energies.

[3] Perissinotto R.M., Verde W.M., Biazussi J.L., Bulgarelli N.A.V., Fonseca W.D.P., de Castro M.S., de Moraes Franklin E., Bannwart A.C. (2021). Flow visualization in centrifugal pumps: A review of methods and experimental studies. J. Pet. Sci. Eng..

[4] Zhou D., Sachdeva R. (2010). Simple model of electric submersible pump in gassy well. J. Pet. Sci. Eng..

[5] Zhou L., Han Y., Lv W., Yang Y., Zhu Y., Song X. (2020). Numerical Calculation of Energy Performance and Transient Characteristics of Centrifugal Pump under Gas-Liquid Two-Phase Condition. Micromachines.

[6] He D., Zhao L., Chang Z., Zhang Z., Guo P., Bai B. (2021). On the performance of a centrifugal pump under bubble inflow: Effect of gas-liquid distribution in the impeller. J. Pet. Sci. Eng..

[7] Shao C., Li C., Zhou J. (2018). Experimental investigation of flow patterns and external performance of a centrifugal pump that transports gas-liquid two-phase mixtures. Int. J. Heat Fluid Flow.

[8] Verde W.M., Biazussi J.L., Sassim N.A., Bannwart A.C. (2017). Experimental study of gas-liquid two-phase flow patterns within centrifugal pumps impellers. Exp. Therm. Fluid Sci..

[9] Zhang J., Cai S., Li Y., Zhu H., Zhang Y. (2016). Visualization study of gas–liquid two-phase flow patterns inside a three-stage rotodynamic multiphase pump. Exp. Therm. Fluid Sci..

[10] Zhao L., Chang Z., Zhang Z., Huang R., He D. (2021). Visualization of gas-liquid flow pattern in a centrifugal pump impeller and its influence on the pump performance. Meas. Sens..

[11] Schäfer T., Bieberle A., Neumann M., Hampel U. (2015). Application of gamma-ray computed tomography for the analysis of gas holdup distributions in centrifugal pumps. Flow Meas. Instrum..

[12] Neumann M., Schäfer T., Bieberle A., Hampel U. (2016). An experimental study on the gas entrainment in horizontally and vertically installed centrifugal pumps. J. Fluids Eng..

[13] Ding L., Shi B., Lv X., Liu Y., Wu H., Wang W., Gong J. (2016). Investigation of natural gas hydrate slurry flow properties and flow patterns using a high pressure flow loop. Chem. Eng. Sci..

[14] Li W., Guo L., Xie X. (2017). Effects of a long pipeline on severe slugging in an S-shaped riser. Chem. Eng. Sci..

[15] Zhou H., Guo L., Yan H., Kuang S. (2018). Investigation and prediction of severe slugging frequency in pipeline-riser systems. Chem. Eng. Sci..

[16] Yin H., Zhou Y., Zhao J., Du Y., An Q., Wang Y., Ma L. (2019). Flow-pattern recognition and dynamic characteristic analysis based on multi-scale marginal spectrum entropy. Appl. Therm. Eng..

[17] Elperin T., Klochko M. (2002). Flow regime identification in a two-phase flow using wavelet transform. Exp. Fluids.

[18] Du M., Jin N.-D., Gao Z.-K., Sun B. (2012). Analysis of total energy and time-frequency entropy of gas–liquid two-phase flow pattern. Chem. Eng. Sci..

[19] Sun B., Erpeng W., Yang D., Bai H., Huang Y. (2011). Time-frequency signal processing for gas-liquid two phase flow through a horizontal venturi based on adaptive optimal-kernel theory. Chin. J. Chem. Eng..

[20] Euh D.J., Song C.-H. (2010). An application of the wavelet analysis technique for the objective discrimination of two-phase flow patterns. Int. J. Multiph. Flow.

[21] Ye J., Guo L. (2013). Multiphase flow pattern recognition in pipeline–riser system by statistical feature clustering of pressure fluctuations. Chem. Eng. Sci..

[22] Zou S., Guo L., Xie C. (2017). Fast recognition of global flow regime in pipeline-riser system by spatial correlation of differential pressures. Int. J. Multiph. Flow.

[23] Lin Z., Liu X., Lao L., Liu H. (2020). Prediction of two-phase flow patterns in upward inclined pipes via deep learning. Energy.

[24] Xu Q., Li W., Liu W., Zhang X., Yang C., Guo L. (2020). Intelligent recognition of severe slugging in a long-distance pipeline-riser system. Exp. Therm. Fluid Sci..

[25] Xu Q., Zhou H., Zhu Y., Cao Y., Huang B., Li W., Guo L. (2020). Study of identification of global flow regime in a long pipeline transportation system. Powder Technol..

[26] Rosa E., Salgado R., Ohishi T., Mastelari N. (2010). Performance comparison of artificial neural networks and expert systems applied to flow pattern identification in vertical ascendant gas–liquid flows. Int. J. Multiph. Flow.

[27] Abbagoni B.M., Yeung H. (2016). Non-invasive classification of gas–liquid two-phase horizontal flow regimes using an ultrasonic Doppler sensor and a neural network. Meas. Sci. Technol..

[28] Ghosh S., Pratihar D., Maiti B., Das P. (2012). Identification of flow regimes using conductivity probe signals and neural networks for counter-current gas–liquid two-phase flow. Chem. Eng. Sci..

[29] Chawla N.V., Bowyer K.W., Hall L.O., Kegelmeyer W.P. (2002). SMOTE: Synthetic minority over-sampling technique. J. Artif. Intell. Res..

[30] Wang S., Liu S., Zhang J., Che X., Yuan Y., Wang Z., Kong D. (2020). A new method of diesel fuel brands identification: SMOTE oversampling combined with XGBoost ensemble learning. Fuel.

[31] Sharma S., Sharma S. (2017). Activation functions in neural networks. Towards Data Sci..

[32] Wang S., Sobecki N., Ding D., Zhu L., Wu Y.-S. (2019). Accelerating and stabilizing the vapor-liquid equilibrium (VLE) calculation in compositional simulation of unconventional reservoirs using deep learning based flash calculation. Fuel.

[33] Phaisangittisagul E. An analysis of the regularization between L2 and dropout in single hidden layer neural network. Proceedings of the 2016 7th International Conference on Intelligent Systems, Modelling and Simulation (ISMS).

[34] He D., Ge Z., Bai B., Guo P., Luo X. (2020). Gas–Liquid Two-Phase Performance of Centrifugal Pump Under Bubble Inflow Based on Computational Fluid Dynamics–Population Balance Model Coupling Model. J. Fluids Eng..

